# Supplementary material for: Application of Fourier Transform Infrared (FT-IR) Spectroscopy, Multispectral Imaging (MSI) and Electronic Nose (E-Nose) for the Rapid Evaluation of the Microbiological Quality of Gilthead Sea Bream Fillets
Source: Foods. 2022 Aug 6;11(15):2356. doi: 10.3390/foods11152356 (PMC9367857; doi:10.3390/foods11152356)
Supplement: Supplementary file 1 [file foods-11-02356-s001.zip › foods-1834713-supplementary.pdf]

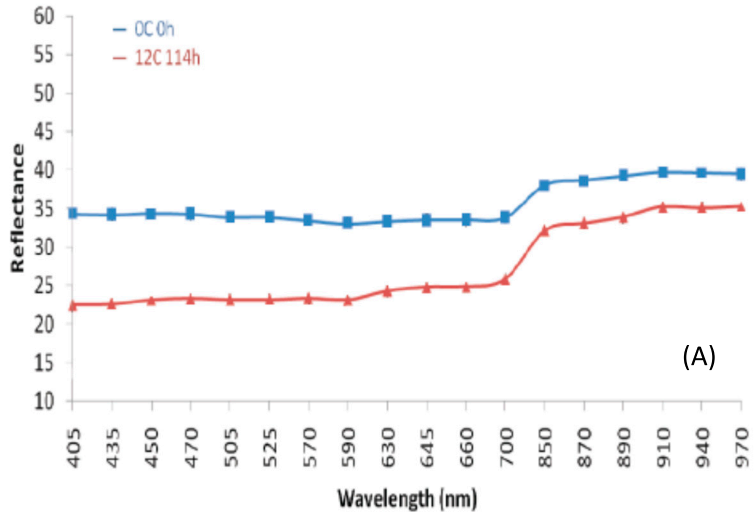

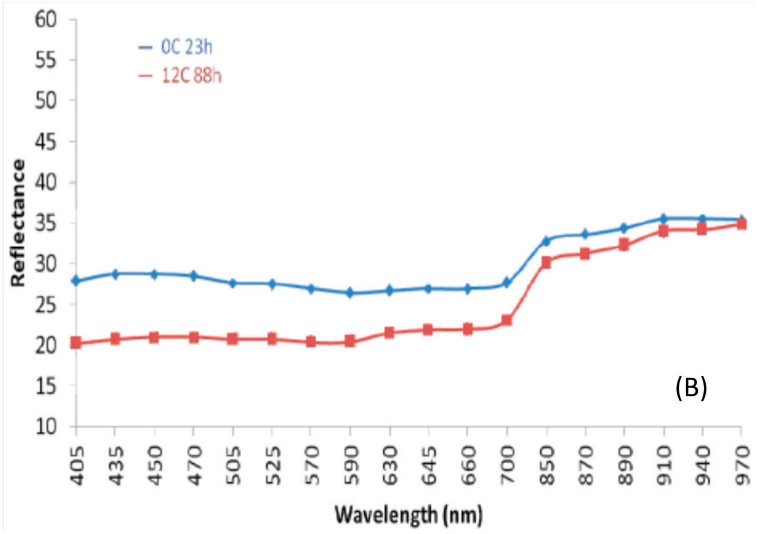

Figure S1. Representative spectra of MSI of fresh (blue line) and spoiled (red line) gilthead sea bream fillets stored under air (A) and under MAP (B).

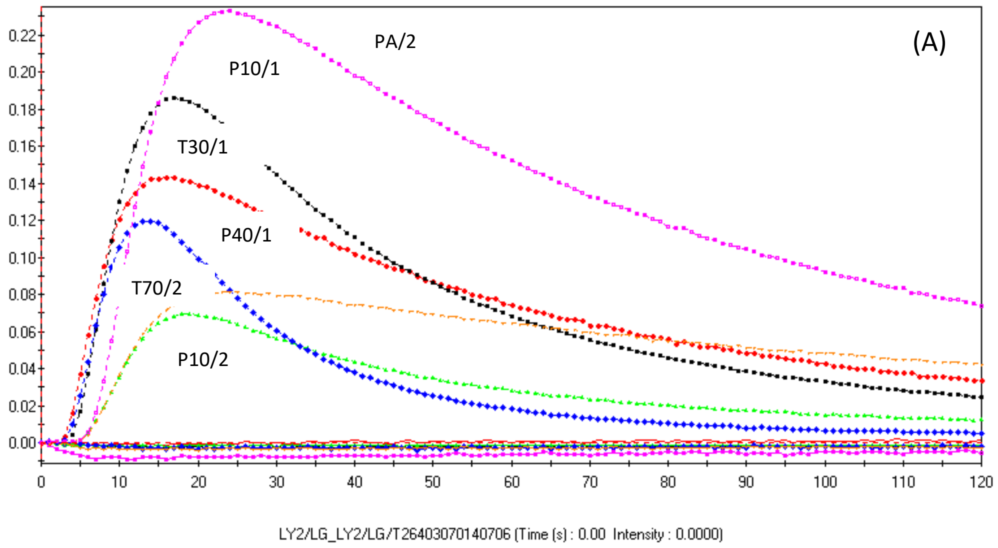

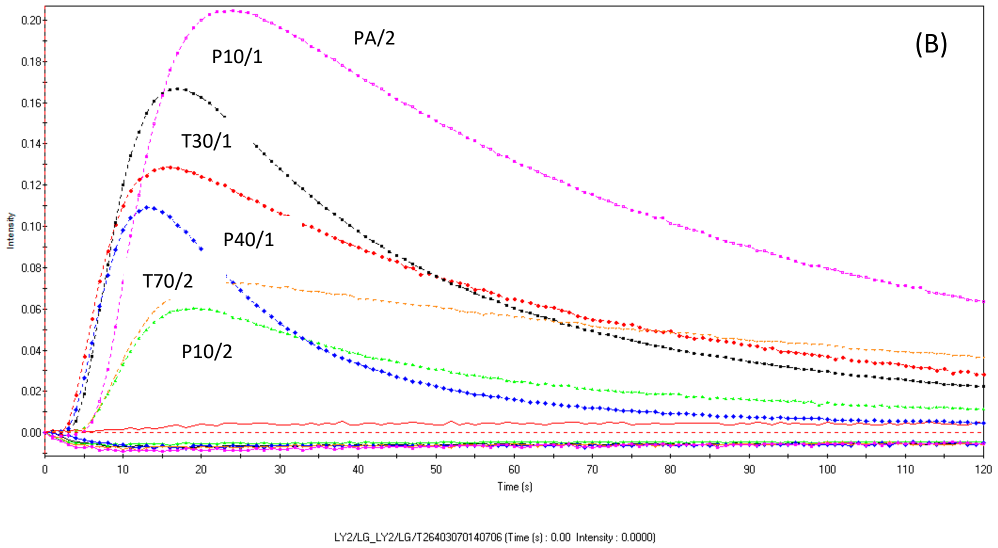

Figure S2. Representative signals of E-nose sensors from fresh (0°C/0 h) (A) gilthead sea bream fillets stored under air and spoiled (0°C/185 h) (B) gilthead sea bream fillets stored under MAP.
